# Pharmacological approaches to the prevention of type 2 diabetes mellitus

**DOI:** 10.3389/fendo.2023.1118848

**Published:** 2023-03-09

**Authors:** Priyanka Majety, Faustina Alejandra Lozada Orquera, Dinesh Edem, Osama Hamdy

**Affiliations:** ^1^ Division of Endocrinology, Diabetes and Metabolism, Virginia Commonwealth University Health System, Richmond, VA, United States; ^2^ Division of Endocrinology, Diabetes and Metabolism, University of Arkansas Medical Center, Little Rock, AR, United States; ^3^ Joslin Diabetes Center, Harvard Medical School, Boston, MA, United States

**Keywords:** type 2 diabetes mellitus, prevention, metformin, prediabetes, remission, pharmacotherapy

## Abstract

About 1 in 10 adults worldwide are estimated to have diabetes mellitus. They are at risk of developing life-threatening complications resulting in reduced quality of life, increased mortality and higher healthcare costs. The ability to prevent or delay type 2 diabetes mellitus (T2DM) by modifying some of its risk factors has been hypothesized for decades. The long and often gradual time-course of increasing dysglycemia prior to diabetes diagnosis suggests that interventions during that period could be effective in preventing T2DM. In addition to lifestyle modifications, certain drugs prevent or slow development of hyperglycemia. Recently, drugs used for obesity management were shown to prevent T2DM. In this review, we discuss various pharmacotherapeutic options for preventing T2DM.

## Introduction

The epidemic of diabetes mellitus and its complications pose major global health threat. The global prevalence of diabetes and impaired glucose tolerance (IGT) quadrupled in the past three decades. This pace of change in diabetes prevalence in many countries has been heightened by rapid urbanization (1, 2). The global prevalence of diabetes was estimated to be 463 million (9.3% of adults 20–79 years of age) and this estimate is projected to rise to 700 million by 2045 (3). Over 90% of diabetes mellitus cases are type 2 diabetes mellitus (T2DM) (4). T2DM is also associated with increased health care cost; estimated to be $850 billion globally (5). The enormous human and financial costs that accompany T2DM, and the challenge of treating it effectively once it is diagnosed, make it an ideal target for prevention.

The ability to prevent or delay T2DM by modifying some of its risk factors has been hypothesized for decades. The long and gradual time-course of increasing dysglycemia prior to T2DM diagnosis suggests that interventions during that period could be effective in prevention of the disease. Substantial progress has been made in recent years in evaluating effective preventive strategies. In addition to lifestyle modifications, certain drugs were shown to prevent or delay development of hyperglycemia. Recently, drugs targeting obesity, have also been studied for T2DM prevention.

In this review, we discuss various pharmacotherapeutic options for preventing T2DM and discuss the evidence behind them as summarized in **Tables 1**, **2**.

**Table 1:** Summary of clinical trials on T2DM prevention with glucose lowering drugs and weight-loss medications.

Study (year)	Eligibility	Number of Subjects	Interventions	Results	Mean or Median Follow up in years	Comments
A) Metformin						
DPP (1996–2001) (6)	IFG and IGT	3234 study participants 531 subjects with IGT	LSM vs. Metformin 850mg BD vs. placebo	LSM group reduced their risk of developing T2DM by 58% and metformin by 31%	2.8	**10-year follow-up (** 7): T2DM incidence was reduced by 34% (24–42) in the lifestyle group and 18% (7–28) in the metformin group compared with placebo. **15 years follow-up (** 8): T2DM incidence was reduced by 27% in the lifestyle intervention group (HR 0.73, 95% CI 0.65-0.83; p<0.0001) and by 18% in the metformin group (0.82, 0.72-0.93; p=0. 001), compared with placebo **22-year follow-up** (9): Relative risk reduction in T2DM by 25% in the lifestyle intervention and by 18% in the metformin group.
IDPP (2006) (10)	IGT	531	LSM vs. Metformin vs. LSM + metformin vs. placebo	Risk reduction for T2DM with LSM was 28.5%, metformin 26.4%, and combination 28.2% compared to placebo	30 months	
CDPP (2001) (11)	IGT	321	LSM vs. Acarbose 50 mg TID vs. metformin 250 mg TID vs. placebo	Annual incidence of T2DM was 11.6% for the control, 8.2% for LSM, 2% for acarbose, and 4.1% for metformin.	3.0	
EDIT (2003) (12)	IGT and IFG	631	Acarbose 50 mg TID vs. metformin 500 mg TID vs. placebo	8% risk reduction with acarbose and 37% with metformin, compared to placebo	6.0	6-year follow-up (13): No differences in relative risk for diabetes with acarbose (1.04, P = 0.81), Metformin (0.99, P = 0.94) or combination therapy (1.02, P = 0.91). In those with IGT at baseline, relative risk was reduced significantly with acarbose (0.66, P = 0.046) but not Metformin (1.09, P = 0.70) or combination therapy (0.72, P = 0.27).
Begum MR et al. (2009) (14)	Nondiabetic –PCOS. Pregnant	29 patients on metformin during pregnancy compared with 30 controls.	Metformin 2 – 2.5 g per day vs. controls	GDM developed in 3.44% of patients on metformin compared to 30% in controls	Gestation period	PCOS use of metformin throughout pregnancy is associated with and might be responsible for a ninefold reduction (30-3.44%) of GDM
Glueck et al. (2002) (15)	Nondiabetic –PCOS. Pregnant	33 patients on metformin compared with 39 controls	Metformin 850 mg TID vs. controls	In PCOS use of metformin is associated with a 10-fold reduction in GDM (31 to 3%)	Gestation period	
Tao Tao et al. (2021) (16)	PCOS + prediabetes (IFG and/or IGT)	150 women with PCOS	Exenatide (10-20μg daily), Metformin (1500-2000 mg daily), or Combination (Exenatide plus Metformin)	Remission rate of prediabetes: Combination group (64%, 32/50) and exenatide group (56%, 28/50) was significantly higher than that of the metformin group (32%, 16/50) (P = .003 and.027, respectively)	12 weeks	
Guardado-Mendoza et al. (2019) (17)	IGT	144	Linagliptin 5 mg + metformin 1700 mg daily + lifestyle (LM group) or metformin 1700 mg daily + lifestyle (M group)	T2DM incidence was higher in M group in comparison to LM group (HR 4.0, 95% CI: 1.24-13.04, p = .020). The probability of achieving normoglycemia was higher in LM group (OR 3.26 CI 95% 1.55-6.84).	2.0	
Daniele G et al. (2020) (18)	History of GDM + IGT or IFG	40	Sitagliptin (100 mg qd), Metformin (850 mg bid) or both (50 + 850 mg bid) for 16 weeks	Among Metformin + sitagliptin women, 33% reverted to normal glucose tolerance (NGT) compared with 14% with Metformin alone and 7% with Sitagliptin (P < 0.05)	16 weeks	
B) Thiazolidinediones						
TRIPOD (2002) (19)	Hispanic women with previous GDM	266	Troglitazone 400 mg/day vs. placebo	55% reduction in the incidence of T2DM in troglitazone arm	2.5	
PIPOD (2006) (20)	Hispanic women with previous GDM	Open label study on 89 women without T2DM in TRIPOD A	Pioglitazone 45 mg/day	Annual T2DM incidence was 4.6%		
ACT NOW (2011) (21)	IGT Mean BMI 33.4 kg/m2	602	Pioglitazone 45 mg/day vs. placebo	Annual incidence rates for T2DM mellitus were 2.1% in the pioglitazone group and 7.6% in the placebo group, and the hazard ratio for conversion to T2DM in the pioglitazone group was 0.28	2.4	Weight gain was greater with pioglitazone than with placebo (3.9 kg vs. 0.77 kg, P<0.001), and edema was more frequent (12.9% vs. 6.4%, P=0.007)
DREAM (2006) (22, 23)	IGT and/or IFG Mean BMI 30.8 kg/m2	5269	Rosiglitazone 8 mg/day vs. placebo and Ramipril 15 mg/day vs. placebo	3-year T2DM incidence: Ramipril: HR 0.91 (0.80–1.03) Rosiglitazone: HR 0.38 (0.33–0.44) HF results: 14 (0·5%) participants in the rosiglitazone group and two (0·1%) in the placebo group developed heart failure (p=0·01).	3.0	Balancing both the benefits and risks suggests that for every 1000 people treated with rosiglitazone for 3 years, about 144 cases of T2DM will be prevented, with an excess of four to five cases of congestive heart failure.
C) Alpha-glucosidase inhibitors						
CDPP	**See above**					
STOP NIDDM (2002) (24)	IGT Mean BMI 31.0 kg/m2	1418	Acarbose 100 mg TID vs. placebo	25% relative risk reduction on acarbose compared to placebo. Absolute risk reduction after 3.3 years was 9.1%.	3.3	Additional studies showed that acarbose was associated with 49% reduction in cardiovascular events (15 vs. 32 subjects; HR 0.51, 95% CI 0.01–0.95, p=0.03). Interpret with caution due to small number of events. NNT 11 for 3.3 years
Ryuzo Kawamori et al. (2010) (25)	IGT	1780	Voglibose 0.2 mg TID vs. placebo	Subjects treated with voglibose had a significantly lower risk for the progression to T2DM than placebo (50/897 vs 106/881: hazard ratio 0.595).	4.0	810 (90%) of 897 patients in the voglibose group had adverse events. Serious adverse events (all one each) in the voglibose group were cholecystitis, colonic polyp, rectal neoplasm, inguinal hernia, liver dysfunction, and subarachnoid hemorrhage.
EDIT	**See above**					
D) Sodium-glucose co-transporter 2 (SGLT2) inhibitors						
Rossing, et al. (2022) (26)	No h/o diabetes and HbA1C less than 6.5%. Mean GFR: 58.7 Mean BMI: 27.4 kg/m2	4003 (1398 [34·9%] from the DAPA-CKD trial and 2605 [65·1%] from the DAPA-HF trial)	Dapagliflozin vs placebo	126 (6·3%) of 2008 patients in the placebo group (event rate 3·9 per 100 patient-years) and 85 (4·3%) of 1995 patients in the dapagliflozin group (event rate 2·6 per 100 patient-years) developed T2DM (hazard ratio 0·67 [95% CI 0·51 to 0·88]; p=0·0040).	1.8	
Weight-loss medications:						
A) Orlistat						
XENDOS (2004) (27)	Obese and normal (79%) or IGT (21%) Mean BMI 37.3 kg/m2	3,305	LSM plus either orlistat 120 mg or placebo, three times daily.	Diabetes risk reduction of 45% compared to placebo	4.0	Overall, 4% of placebo patients and 8% of orlistat patients withdrew from the study because of adverse events, primarily GI due to gastrointestinal events.
B) Phentermine-Topiramate						
CONQUER (2011) (28)	Overweight or obese adults and two or more comorbidities (hypertension, dyslipidemia, T2DM or prediabetes, or abdominal obesity) Mean BMI 36.2 kg/m2 (low dose) 36.6 kg/m2 (high dose)	2487 (1684 (68%) had IGT or IFG)	Placebo vs once-daily phentermine 7·5 mg plus topiramate 46·0 mg vs once-daily phentermine 15·0 mg plus topiramate 92·0 mg.	the relative risk (vs placebo) was 0·78 (0·40–1·50) with phentermine 7·5 mg plus topiramate 46·0 mg, and 0·47 (0·25–0·88) with phentermine 15·0 mg plus topiramate 92·0 mg	1.0	
C) Lorcaserin						
CAMELLIA-TIMI 61 (29)	BMI ≥27 kg/m^2^ with or at high risk for atherosclerotic vascular disease; Age ≥ 40 years	Total N: 12,000 At baseline, 6816 patients (56.8%) had T2DM, 3991 (33.3%) prediabetes, and 1193 (9.9%) normoglycemia.	Lorcaserin 10mg BID or placebo	Lorcaserin reduced T2DM incidence by 19% (HR, 0.81; 95% CI: 0.66– 0.99) in patients with prediabetes and by 23% in patients without diabetes (HR 0·77, 95% CI: 0·63–0·94).	3.3	

DPP, Diabetes Prevention Program; IDPP, Indian Diabetes Prevention Program; CDPP, Chinese Diabetes Prevention Program; EDIT, Early Diabetes Intervention Trial; STOP NIDDM, Study to prevent non-insulin dependent diabetes; TRIPOD, Troglitazone in Prevention of Diabetes; PIPOD, Pioglitazone in Prevention of Diabetes; ACT NOW, Actos Now for the prevention of diabetes; DREAM, Diabetes Reduction Approaches with Ramipril and Rosiglitazone Medications; LSM, Lifestyle modifications; IGT, Impaired glucose tolerance; IFG, Impaired fasting glucose; GDM, Gestational diabetes mellitus; BD, Twice daily; TID, Thrice daily; CKD, chronic kidney disease; GFR, Glomerular Filtration Rate; XENDOS: XENical in the prevention of diabetes in obese subjects study, CONQUER: Effects of low-dose, controlled-release, phentermine plus topiramate combination on weight and associated comorbidities in overweight and obese adults, CAMELLIA-TIMI: Effect of lorcaserin on treatment and prevention of type 2 diabetes in overweight and obese patients.

**Table 2:** Summary of evidence on T2DM prevention with medications promoting both glucose-lowering and weight-loss.

Study (year)	Eligibility	Number of Subjects	Interventions	Results	Mean or Median Follow up in years	Comments
A) Glucagon-like peptide-1 agonists						
Rosenstock, et al. (2014) (30)	Obese, subjects, Mean BMI: 39.6 kg/m2 and IGT or IFG	152	Placebo vs Exenatide plus LSM	IGT or IFG normalized at end point in 77% of exenatide vs 56% of placebo subjects.	24 weeks	
Le Roux et al. (2017) (31)	Prediabetes plus BMI of at least 30 kg/m2 or 27 kg/m2 with comorbidities.	2254	Once-daily subcutaneous liraglutide 3·0 mg or matched placebo plus LSM	In the liraglutide group 26 individuals (2%) of 1472 were diagnosed with T2DM vs 46 (6%) of 738 in the placebo group. The time to onset of T2DM over 160 weeks among all randomized individuals was 2·7 times longer with liraglutide than with placebo (95% CI 1·9 to 3·9, p<0·0001)	160 weeks	
Garvey, et al. (2022) (32) Wilding, et al. (2021) (33) - STEP 1	BMI ≥ 30 kg/m2 or ≥ 27 kg/m2 with at least one weight-related complication, excluding T2DM	1961	Once a week 2.4mg semaglutide vs placebo	Risk scores decreased from 18% to 7% with semaglutide, and 18% to 16% with placebo (61% vs. 13% reduction [p<0.01]	68 weeks	10-yr T2D risk was calculated *post hoc*, from STEP 1 (68 weeks) and STEP 4 (20-wk run-in on semaglutide, 48-wk randomized withdrawal)
Garvey, et al. (2022) (32) Rubino, et al. (2021) (34)- STEP 4	BMI ≥ 30 kg/m2 or ≥ 27 kg/m2 with at least one weight-related complication, excluding T2DM	902	Once a week 2.4mg semaglutide vs placebo	Risk score reduction with semaglutide occurred during wks 0-20, from 21% to 8% but increased to 15% with switch to placebo (32% reduction vs. 41% increase [p<0.01]	20-wk run-in on sema, 48-wk randomized withdrawal	As above
B) Tirzepatide						
SURMOUNT-1 (35)	BMI ≥ 30 kg/m2 or ≥ 27 kg/m2 with at least one weight-related complication, excluding T2DM	At baseline, 40.6% (n=1032 out of 2539) of subjects had prediabetes	Tirzepatide (5 mg, 10 mg, or 15 mg) or placebo	95.3% of the participants with prediabetes at baseline in the treatment groups had reverted to normoglycemia, as compared with 61.9% of participants in the placebo group.	1.4	

BMI, body-mass index; LSM, lifestyle modifications; IGT, impaired glucose tolerance; IFG, impaired fasting glucose; GDM, gestational diabetes mellitus; T2DM, type 2 diabetes mellitus; STEP, Semaglutide Treatment Effect in People with Obesity; SURMOUNT: Efficacy and safety of Tirzepatide once weekly in participants without type 2 diabetes who have obesity or are overweight with weight- related comorbidities.

## Glucose-lowering medications

### Metformin

Metformin, a biguanide, primarily decreases hepatic glucose production by inhibiting gluconeogenesis. It enhances peripheral insulin sensitivity in the skeletal muscle by increasing insulin receptor tyrosine kinase activity and glucose transporter (GLUT)-4 translocation to the cell membrane (36). Metformin also improves beta-cell responsiveness to a glucose load through correction of glucotoxicity (37).

The Diabetes Prevention Program (DPP) study was a multi-center trial which enrolled 3,234 subjects with prediabetes and randomized them to either intensive lifestyle intervention (intended to achieve 7% body weight loss), metformin (850mg twice daily) or standard lifestyle recommendations (38). Participants in the study were overweight or obese (mean BMI 34 kg/m^2^), mostly middle-aged adults with IGT or impaired fasting glucose (IFG) with values between 95-126 mg/dL. In the ensuing 2.8 years, T2DM incidence was reduced by 58% with lifestyle intervention and by 31% with metformin compared to placebo (6). It is interesting to note that genome-wide association studies in the DPP cohort revealed novel ethnic-specific associations with metformin response and may have implications for individualized therapy (39).

A secondary analysis of the DPP study was performed in subjects with history of gestational diabetes (GDM). Women with history of GDM were compared with women with previous live birth but without GDM history. It was found that progression to T2DM is more common among women with history of GDM compared with those without it, despite equivalent degrees of IGT at baseline. Both lifestyle modifications (LSM) (53% reduction in T2DM incidence) and metformin (50% reduction in T2DM incidence) were highly effective in delaying T2DM in women with IGT and history of GDM (40).

Eighty-eight percent of the surviving DPP cohort enrolled in a long-term follow up study, the Diabetes Prevention Program Outcomes Study (DPPOS). During the DPPOS, unmasked metformin was continued as a study intervention in the original metformin group. T2DM incidence was reduced by 34% in the lifestyle group and 18% in the metformin group compared to placebo in the following 10 years (7). Significant reduction in T2DM incidence persists in the metformin group at 15-year (relative risk reduction/RRR 18%; *p*=0. 001) (8) and 22-year follow up (RRR 18%) (9).

Similarly, The Indian Diabetes Prevention Program (IDPP) randomized 531 Asian Indian subjects with IGT (mean BMI 25.8 kg/m2) to 4 groups: LSM or metformin (250-500mg twice daily) or LSM plus metformin or control group (standard healthcare advice). The median follow-up period was 30 months. The 3-year cumulative incidences of diabetes were 39.3%, 40.5%, 39.5% and 55.0%, respectively. The RRR was 26.4% with metformin (95% CI 19.1–35.1, p=0.029) and 28.2% with LSM plus metformin (95% CI 20.3–37.0, p=0.022), as compared to the control group (10).

The Chinese Diabetes Prevention Program (CDPP) evaluated the preventive effect of lifestyle intervention with diet and exercise, acarbose, and metformin on T2DM progression in 321 subjects with IGT. Annual T2DM incidence was 11.6, 8.2, 2.0, and 4.1% in the control, lifestyle intervention, acarbose, and metformin groups, respectively (11).

The Early Diabetes Intervention Trial (EDIT) analyzed the effects of metformin and acarbose in T2DM prevention in 631 subjects with IFG. At three years, there was an 8% risk reduction with acarbose and 37% with metformin, compared to placebo (12) but there was no difference in the relative risk for T2DM at six-year of follow-up. For patients with IGT at baseline, the RRR was significant with acarbose (0.66) but not with metformin (1.09), suggesting different effects of different therapies in subjects with IGT and IFG (13).

A study by Begum et al. (2009) showed that occurrence of GDM among women with polycystic ovarian syndrome (PCOS) was significantly lower in the metformin treatment group with only one subject (3.44%) vs nine of the 30 pregnancies (30%) without metformin (14). Similarly, Glueck et al. (2002) reported the odds ratio for GDM in women with metformin vs those without metformin was 0.093 (95% CI: 0.011 to 0.795) (15).

Recent studies showed promising effects of metformin in combination with glucagon-like peptide-1 (GLP-1) receptor agonists (16) and dipeptidyl peptidase 4 (DPP-4) inhibitors (17, 18) for T2DM prevention.

According to the American Diabetes Association (41), metformin should be considered for T2DM prevention in adults with BMI ≥35 kg/m2, age ≤ 60 years, higher fasting plasma glucose (≥ 110 mg/dL), and higher A1C (≥ 6.0%), and in women with prior GDM.

### Thiazolidinediones

Thiazolidinediones (TZDs) are insulin sensitizers. They activate gamma isoform of peroxisome proliferator-activated receptor (PPAR γ), enhance glucose uptake by skeletal muscles and adipocytes, improve insulin sensitivity and consequently improve pancreatic beta-cell function (42, 43).

The Troglitazone in Prevention of Diabetes (TRIPOD) study (19) compared Troglitazone with placebo in 266 nondiabetic Hispanic women (mean age 34.6 years; mean BMI 30.5 kg/m^2^) with previous GDM, about 70% of whom had IGT at entry into the trial. Troglitazone treated group had a 55% reduction in the incidence of T2DM over 2.5 years. The drug was recalled before the planned study-end because of reports of hepatic failure.

The Pioglitazone in Prevention of Diabetes (PIPOD) study (20) was an open-label follow-up of 89 women from TRIPOD who had not developed diabetes (A1C <7%). These participants showed an average rate of diabetes of 4.6% per year during treatment with pioglitazone for three years, which was much lower compared with the rate of 12.1% per year that was observed during placebo treatment in the TRIPOD study.

Actos Now for the Prevention of Diabetes (ACT NOW) study (21) was an RCT conducted to examine the effectiveness of pioglitazone in preventing T2DM among 602 subjects (mean age 52 years; mean BMI 34 kg/m^2)^ with IGT. The annual rate of T2DM was 7.6% in placebo-treated vs. 2.1% in pioglitazone treated subjects (HR 0.28, p<0.0001) over 2.4 years.

Concerns over adverse effects of TZDs has dampened the enthusiasm to use pioglitazone for T2DM prevention. Undesirable effects of TZDs include fluid retention, increased risk of heart failure, weight gain and loss of bone density increasing fracture risk.

### Alpha-glucosidase inhibitors

These drugs act by competitively inhibiting alpha- glucosidase enzyme and decreasing carbohydrate absorption from the small intestine, thus reducing postprandial glucose levels (44).

The Study TO Prevent Non-Insulin Dependent Diabetes Mellitus (STOP-NIDDM) (24) evaluated the effects of acarbose in delaying progression of IGT to T2DM in 1429 subjects. Over the 3.3-year follow-up period, there was a 25% reduction in the incidence of T2DM in the acarbose group compared to placebo. Furthermore, acarbose significantly increased the regression of IGT to normal glucose tolerance (HR 1.42, 95% CI 1.24–1.62; *p*<0·0001). About one-quarter of the cohort did not complete the study, and this was attributed to the gastrointestinal side effects of acarbose.

Kwawamori et al., randomized 1780 Japanese subjects with IGT to either receiving voglibose 0.6 mg/day or placebo. At the end of the study (11 months; ended early due to efficacy) the T2DM hazard ratio (voglibose vs. placebo) was 0.60 (95% CI 0.43–0.82) (25).

### Sodium-glucose co-transporter-2 inhibitors

The SGLT2 is expressed in the proximal tubule and mediates reabsorption of approximately 90 percent of the filtered glucose load to the kidneys. SGLT2 inhibitors promote renal excretion of glucose and thereby modestly lower elevated blood glucose levels (45).

Pre-specified pooled analysis (26) of the DAPA-CKD and DAPA-HF trials suggests that dapagliflozin (10 mg daily) may reduce new-onset T2DM compared with placebo (HR 0·67 [95% CI 0·51–0·88]). In pooled analysis of selected 4,003 participants who had no previous diagnosis of T2DM (mean age 63 years, mean A1C 5.7%), dapagliflozin reduced new-onset T2DM incidence (defined as having A1C ≥6.5% on two consecutive follow-up visits, or a clinical diagnosis of T2DM that led to initiation of a glucose-lowering agent) by approximately one-third. The overall incidence of new-onset T2DM was 2.6 events per 100 patient-years in the dapagliflozin group vs 3.9 events per 100 patient-years in the placebo group (HR 0.67; 95% CI 0.51–0.88; *p*=0.0040). Treatment was predominantly beneficial in participants with prediabetes. Possible mechanisms include protection of pancreatic beta-cells from glucotoxicity, weight loss, and improvement in hepatic insulin sensitivity. Improvement in CKD and heart failure may have contributed to insulin sensitivity (26). A prospective RCTs is needed to confirm whether dapagliflozin truly prevents T2DM.

Although empagliflozin did not demonstrate significant benefit for T2DM prevention among patients with heart failure and prediabetes in EMPEROR-Preserved study (46) (HR: 0.84; 95% CI 0.65–1.07) and EMPEROR-Reduced study (47) (HR: 0.86; 95% CI 0.62–1.19), the hazard ratios were consistent with potential benefit. A recent meta-analysis of four RCTs showed that SGLT2 inhibitors (empagliflozin and dapagliflozin) were significantly associated with a lower risk of new-onset diabetes (relative risk, 0.79; 95% CI, 0.68-0.93) (48). This meta-analysis included 5655 participants with pre-diabetes. The relative risks of new-onset diabetes in dapagliflozin and empagliflozin were 0.68 (95% CI, 0.52-0.89) and 0.87 (95% CI, 0.72-1.04), respectively.

## Weight-loss medications

### Orlistat

Orlistat acts by reversibly inhibiting gastric and pancreatic lipases (49). It also increases postprandial glucagon-like peptide 1 (GLP-1) levels. In a pooled analysis by Heymsfield et al. (50) orlistat compared to placebo, reduced 2-year cumulative diabetes incidence by 61% (7.6% in the placebo group vs. 3.0% in the orlistat group) among those with IGT. However, due to the side effects of orlistat, only 69% of the participated subjects completed the study.

Xenical in the Prevention of Diabetes in Obese Subjects (XENDOS) study (27) was a 4-year, double-blind, prospective study where 3,305 obese subjects with prediabetes were randomized to either lifestyle intervention plus orlistat 120 mg or plus placebo. It showed that the cumulative incidence of T2DM was 9.0% with placebo and 6.2% with orlistat, corresponding to a risk reduction of 37.3% (*p*=0.0032). Independent of orlistat or placebo treatment, the relative risk of developing T2DM was greater in patients with IGT, men, older subjects, and subjects with a higher BMI.

### Phentermine/topiramate

Phentermine is a sympathomimetic drug, which stimulates norepinephrine release in the hypothalamus, suppresses appetite and increases satiety. Topiramate’s action is not well understood but hypothesized to work on alpha-amino-3-hydroxy-5-methyl-4-isoxazolepropionic acid (AMPA) receptors and kainite receptors to reduce food cravings, and on Gamma-aminobutyric acid (GABA) receptors to increase energy expenditure (51).

The CONQUER study evaluated the efficacy of phentermine/topiramate combination as an adjunct to LSM for weight loss and metabolic risk reduction in individuals who are overweight and obese, with two or more risk factors (28). They enrolled 84% of subjects without T2DM at baseline. In this population, development of T2DM was less in phentermine 15 mg/topiramate ER 92 mg group compared to placebo after 56 weeks of intervention (1.7% versus 3.6%; HR, 0.47; 95% CI, 0.25–0.88).

In the long-term follow up study, SEQUEL, a total of 78.1% of subjects in the original CONQUER study continued to take blinded medication over 108 weeks. The annual incidence rates for progression to T2DM were 0.9% in high dose phentermine/topiramate group and 3.7% in the placebo group (*P*=0.008) (52). These studies were not performed to assess prevention of T2DM as a primary outcome.

A subgroup analysis of the CONQUER study including subjects with prediabetes and/or metabolic syndrome at baseline showed that the annual incidence of T2DM was 1.3% for high dose phentermine/topiramate and 6.1% in placebo (53).

### Naltrexone/bupropion

Bupropion is a norepinephrine and dopamine reuptake inhibitor which stimulates proopiomelanocortin (POMC) neurons in the hypothalamus, with a downstream effect of increased satiety. Naltrexone prevents rebound inhibition of POMC neurons by β-endorphin, and synergistically works with bupropion to increase satiety (51).

There were four Contrave Obesity Research (COR) studies looked at the weight loss effects of Naltrexone/Bupropion (NB): the COR-I, COR-II, COR-BMOD (behavior modification), COR-Diabetes (54–57). None of these studies were designed to analyze progression of IGT to T2DM, but COR-I study (54) showed significant decrease in fasting plasma glucose in response to naltrexone SR 32 mg/bupropion SR 16 mg combination treatment. COR-Diabetes study (57) including subjects with T2DM reported improvements in glucose homeostasis, resulting in A1C reduction of 0.6% in the NB group, and the A1C goal of ¾7% was achieved in 44% in NB group vs 26% in placebo group.

Further longer-term studies are needed to establish the effect of this medication in T2DM prevention.

## Drugs promoting glucose-lowering and weight-loss

### Glucagon-like peptide 1 receptor agonists

Glucagon-like peptide 1 receptor agonists (GLP-1 RA) mimic the action of endogenous GLP-1 in enhancing glucose-dependent insulin secretion and suppress glucagon production from pancreatic alpha cells and thus used to treat T2DM. Apart from glycemic control, they are also thought to reduce neuroinflammation, promote nerve growth, improve cardiac function, suppress appetite, delay gastric emptying, regulate lipid metabolism and reduce fat deposition (58).

In a study (30) of 152 obese patients (mean age 46 years; mean BMI 39.6 kg/m2) with and without prediabetes (IGT or IFG), blood glucose was normalized in 77% and 56% after exenatide and placebo, respectively. There was a significantly higher weight loss with exenatide of 5.1 kg vs 1.6 kg with placebo.

In the SCALE study Obesity and Prediabetes trial (31), 2,254 adults with prediabetes and BMI ≥30 kg/m^2^ or ≥27 kg/m^2^ with comorbidities (mean age 47 years, mean BMI 39 kg/m^2^) were randomized to receive liraglutide 3mg/day vs a matched placebo, as an adjunct to LSM. Time to onset of diabetes in the treatment group was 2.7 times longer with liraglutide compared to placebo (95% CI 1·9 to 3·9, p<0·0001; HR 0.21, 95% CI 0.13–0.34). However, about half the participants withdrew from the study. Based on an analysis imputing the missing data, T2DM incidence was reduced by 66% (HR 0.34, 95% CI 0.22–0.53) with liraglutide vs 36% with placebo.

Recently, the effect of semaglutide in T2DM prevention was assessed in comparison to placebo in overweight or obese individuals in the Semaglutide Treatment Effect in People with Obesity (STEP) studies. In a *post-hoc* analysis (32) of the STEP 1 (33) (68 weeks) and STEP 4 (34) (20-week run-in on semaglutide, 48-week randomized withdrawal) trials, it was shown that semaglutide 2.4 mg could reduce the 10-year risk of progression to T2DM by 61%, regardless of the initial glycemic status (vs 13% reduction in the placebo group (*p*<0.01). In this analysis, the 10-year risk of T2DM was calculated using Cardiometabolic Disease Staging (CMDS). Most of the risk reduction occurred during the initial weeks (0-20 weeks), from 21% to 11%. Risk scores further decreased with continued semaglutide (weeks 20-68) but increased with switch to placebo (32% reduction vs. 41% increase, *p*<0.01).

### Dual glucose-dependent insulinotropic polypeptide and GLP-1 receptor agonist

Tirzepatide, the dual GIP/GLP-1 receptor agonist was recently approved by the FDA in May 2022 for treatment of T2DM. In the 72-week SURMOUNT-1 trial (35), 40.6% of the patients had prediabetes at baseline. At the end of the trial, 95.3% of those participants reverted back to normoglycemia, as compared with 61.9% of participants in the placebo group. There was also a notable decrease in the fasting insulin levels in the treatment groups. This is probably attributed to the significant weight loss that was seen in association with tirzepatide (15-20.9% weight loss), however future dedicated studies are still needed to confirm the role of tirzepatide for T2DM prevention.

## Other medications

### Renin angiotensin aldosterone system blockade

Various trials (23, 59–65) suggested that RAAS inhibition may reduce the incidence of new onset T2DM in patients with or without hypertension or at high risk of T2DM. The risk reduction was explained by hemodynamic effects such as improved delivery of insulin and glucose to the peripheral skeletal muscle, non-hemodynamic effects, including direct effects on glucose transport and insulin signaling pathways, which collectively decrease insulin resistance.

A meta-analysis (66) of new-onset T2DM in select comparative outcome trials involving the use of RAAS blockade vs non-RAS blockade showed a lower risk ratio (RR 0.78; 95% CI 0.74-0.88). However, most of these trials did not include T2DM incidence as the pre-specified end point.

### Vitamin D

The Vitamin D and Type 2 Diabetes (D2d) Study (67), randomly assigned 2,423 adults with prediabetes to receive 4,000 units of vitamin D3 per day or placebo, regardless of their baseline vitamin D status. After a median follow-up of 2.5 years, T2DM occurred in 293 participants in the vitamin D group, and 323 in the placebo group (9.39 and 10.66 events per 100 person-years, respectively). The hazard ratio for vitamin D compared with placebo was 0.88 (95% CI, 0.75 to 1.04; *p*=0.12), failing to show a significant decrease in T2DM incidence among the study population.

However, subsequent meta-analyses of studies in patients with pre-diabetes demonstrated that vitamin D supplementation at moderate to high doses significantly reduced the incidence risk of T2DM (68, 69). A secondary analysis of the D2d study showed that participants who maintained higher intratrial serum vitamin D levels during follow-up had a reduced risk of diabetes (70).

### Testosterone

Men who are overweight frequently have low serum testosterone (T) concentrations, which is in turn associated with increased risk of T2DM (71).

T4DM (72) was a randomized, placebo-controlled trial, which included 1,007 men who were enrolled in lifestyle intervention program and randomly assigned to receive T or placebo. At 2 years, 21% of the subjects in the placebo group failed OGTT vs 12% in the T group (RR 0.59, CI 0.43-0.80, *p*=0.007). Treatment effect was independent of baseline serum testosterone level. So far, testosterone use is not approved for T2DM prevention or treatment.

## Conclusion

The rates of T2DM have been rising, parallel to the rates of obesity, largely due to sedentary lifestyle and increased access to highly obesogenic foods. T2DM is one of the leading risk factors for cardiovascular mortality.

Trials involving lifestyle therapies (38, 73, 74) have shown that 5-10% weight loss in obese and overweight individuals with prediabetes is effective in preventing or delaying development of T2DM. Bariatric surgery (75) is particularly effective in preventing T2DM. However, majority of patients are unable to sustain lifestyle changes (76) or accept bariatric surgery as a preventive tool. Appropriate pharmacotherapy is an attractive option to prevent or delay development of T2DM. Metformin should be considered for T2DM prevention in adults with BMI ≥35 kg/m2, age ≤ 60 years, higher fasting plasma glucose (≥ 110 mg/dL), and higher A1C (≥ 6.0%), and in women with prior GDM (41).

Newer therapies like GLP-1 receptor agonists or dual GIP/GLP-1 receptor agonists have unique ability to suppress appetite, and improve pancreatic insulin production, resulting in profound weight loss, improved insulin sensitivity. Although there are several emerging data to show benefits of these therapies in preventing T2DM, further randomized prospective studies are required.

## Author contributions

PM and FO contributed to the conception and design of the manuscript. PM and FO wrote the first draft of the manuscript. DE and OH wrote sections of the manuscript. OH edited the manuscript. All authors contributed to the article and approved the submitted version.
